# Corneal biophysical changes after upper eyelid blepharoplasty and ptosis surgery: a review

**DOI:** 10.1186/s12886-023-03010-3

**Published:** 2023-06-06

**Authors:** Mostafa Heidari, Ali A. Haydar, Mohammad Taher Rajabi, Seyed Mohsen Rafizadeh

**Affiliations:** Farabi Eye Research Center, Department of Oculofacial Plastic and Reconstructive Surgery, Farabi Eye Hospital, Tehran University of Medical Sciences, South Kargar Street, Qazvin Square, Tehran, 1336616351 Iran

**Keywords:** Blepharoplasty, Ptosis, Cornea, Topography

## Abstract

Upper eyelid surgeries, such as blepharoplasty and ptosis correction, are commonly performed procedures worldwide. This review examines the effects of these surgeries on ocular properties and visual function. A search of the PubMed and Google Scholar databases was conducted to identify relevant articles published after 2000. The results demonstrate that the ocular and adnexal organs function as a unified visual system, with changes in one component affecting the functions of others. Eyelid surgery can alter ocular properties and functions by modifying retinal lighting and ocular optics. These alterations can affect intraocular pressure estimation, corneal curvature, corneal epithelial thickness, refractive power of the cornea, and intraocular lens calculation. Additionally, eyelid surgery can exacerbate dry eye symptoms and impact contrast sensitivity, which is a significant factor in visual quality. Therefore, understanding these interactions is crucial before performing eyelid surgery and during follow-up. This review summarizes recent literature on the effects of upper eyelid surgery on corneal properties and visual function, emphasizing the importance of considering these factors when planning or undergoing such procedures.

## Background

Eyelid surgeries, including blepharoplasty and ptosis repair, are among the most performed orbital and cosmetic procedures worldwide. In the United States alone, over 200,000 blepharoplasties are conducted annually [1]. Patients with heavy and structurally lax eyelids may experience increased pressure on the globe, which can affect various ocular measurements such as anterior corneal curvature and epithelial thickness, corneal refractive power, and intraocular lens (IOL) calculations [2–7]. For instance, severe ptosis correction has been found to induce significant changes in corneal spherical and cylindrical power, corneal aberrations, and contrast sensitivity, among other factors [2, 3, 8, 9]. Furthermore, upper eyelid blepharoplasty and blepharoptosis repair can lead to varying degrees of dry eye that may persist for several months after surgery [10, 11]. The impact of these factors on other ocular and orbital therapeutic and cosmetic procedures can be decisive. As such, it may be advisable to perform orbital and cosmetic surgery prior to intraocular surgery to achieve more predictable outcomes with minimal manipulation. However, it is important to note that some surgeons often delay eyelid surgery until after intraocular surgery because the use of an eyelid speculum can stretch the eyelids and orbicularis and cause ptosis.

Given the increasing number of orbital and cosmetic procedures being performed worldwide as well as a growing body of literature examining their effects on visual function, it is imperative that we review existing research to better understand how these surgeries impact ocular surface characteristics, corneal properties, and visual function while also identifying areas where further investigation is needed.

## Methods

We conducted a comprehensive literature search using the PubMed and Google Scholar databases. Our search strategy included these keywords: blepharoplasty, blepharoptosis, ptosis, cornea, corneal topography, dry eye, astigmatism, and tear. We limited our search to English articles published after the year 2000 and carefully selected relevant studies for inclusion in this narrative review. Our search yielded a total of forty original articles, one review article, and one case report. The data extracted from these sources were meticulously analyzed and presented in two tables (Table 1 and Table 2), organized according to the type of surgery performed.

**Table 1:** Summaries of clinical articles reporting the effect of blepharoplasty on corneal biophysics

Author (year)	Aim	Design	Intervention	Methods	Cases (eyes)	Mean age	Outcomes
Saadat and Dresner (2004) [12]	To assess the safety of blepharoplasty in patients with preop dry eyes	Retrospective	Upper blepharoplasty: skin and fat excision. No orbicularis was excised to preserve innervation Lower blepharoplasty: transconjunctival approach for fat removal	Full history, ocular examination, basic Schirmer test with anesthesia	67	64	8% reported worsening in the severity of their dry eyes, 8% improvement, 83% no change By preserving the orbicularis muscle and its innervation, the dynamics of eyelid closure, tear pumping, and tear distribution are not affected
Kim et al. (2007) [13]	To evaluate the effect of upper eyelid surgery on ocular surface sensation and tear production	Prospective	Blepharoplasty: excision of skin and orbicularis muscle flap Ptosis: orbital septum opened and levator aponeurosis advanced or resected	Cochet–Bonnet esthesiometer, Schirmer 1 test without anesthesia	11 (21)	62	A significant temporary decrease in ocular surface sensation that returns to baseline after 1 month
Zinkernagel et al. (2007) [14]	To compare the effects of different upper eyelid procedures on corneal topography	Prospective	Skin-only blepharoplasty, blepharoplasty with reduction of the medial fat pad, blepharoplasty with reduction of the entire fat pad, and levator advancement	Computed corneal topography before surgery and at 3 months	43 (82)	59	Statistically significant correlation between the severity of upper eyelid abnormality and topographical corneal changes after surgery Changes in astigmatism were greater when large fat pads were reduced Postop astigmatic axis changes were not systematic
Rogers et al. (2012) [15]	To assess the effect of upper eyelid blepharoplasty on CS	Prospective	Routine upper eyelid blepharoplasty under local anesthetic	Pelli-Robson chart at 1 m, VA, and automated 60:4 visual field	14 (28)	63.5	Significant increase in log CS from 1.49 to 1.64
Kim et al. (2013) [2]	To assess CS and VA after upper eyelid blepharoplasty	Prospective	Excess skin, orbicularis, and fat pad excision	CS was measured by an automated Contrast Glaretester. HOA by KR-1W Wavefront Analyser. Lash ptosis was measured using a 4-point rating scale	16 (22)	47.4	CS significantly increased in every spatial frequency and light condition HOA (total HOA, 3^rd^ and 4^th^ order, trefoil, coma, and second astigmatism decreased significantly. Lash ptosis also decreased after. Corneal topography showed no difference
Dogan et al. (2015) [16]	To evaluate corneal parameters obtained by Scheimpflug imaging after blepharoplasty	Prospective	Excess skin and fat pad excision	Preop and postop (3rd month) Sheimpflug imaging: CCT, ACD, steepest keratometry, astigmatic power vectors	30 (60)	56.5	The only parameter that was significantly different was the steepest keratometry in patients with preop MRD1 < 2.5 mm Other parameters showed no differences
Simsek et al. (2015) [9]	To determine any change in corneal astigmatism and VA changes following upper blepharoplasty	Prospective	Routine upper eyelid blepharoplasty surgery	Pentacam and VA before, 1 and 3 months postop	23 (43)	46.3	Statistically significant astigmatic changes (0.15 D); but clinically insignificant VA changes. No significant change in astigmatism axis was detected
An et al. (2016) [17]	To assess the effects of upper lid blepharoplasty on visual quality	Prospective	Excess skin, orbicularis and fat pad excision	CS: Vector Vision CSV-1000 chart; levator function test; Lash ptosis: 4-point rating scale	39 (73)	62.6	A significant increase in CS under scotopic and photopic conditions were found Lash ptosis also improved
Kim et al. (2016) [18]	To analyze corneal curvature changes after upper eyelid surgery, and to compare the effects of different upper eyelid procedures on corneal curvature	Prospective	Blepharoplasty: excision of redundant skin orbicularis muscle and fat Ptosis: levator resection	Corneal topography before surgery, and at 6 weeks	34 (50)	57.9	Levator resection showed greater changes of corneal curvature (central corneal power and astigmatism) than blepharoplasty The advanced aponeurosis technique may have a greater effect on the lid/cornea interface
Rymer et al. (2017) [11]	To evaluate the effects of Muller’s muscle-conjunctival resection (MMCR) on ocular surface and dry eye symptoms	Prospective	Bilateral upper eyelid skin excision with MMCR or skin-only excision	Salisbury Eye Evaluation Questionnaire, Schirmer’s test, TBUT, fluorescein and rose Bengal staining	46 (92)	62	No changes were seen in patients who underwent blepharoplasty alone in the questionnaire scores. No changes were found in tear production profiles in the blepharoplasty-only group. Addition of MMCR to upper eyelid blepharoplasty did not worsen the dry eye profile
Mohammed (2018) [19]	Study the impact of orbicularis strip excision during upper blepharoplasty on postop dry eye symptoms	Interventional comparative	Upper blepharoplasty ± orbicularis excision	Corneal and tear film, TBUT and Schirmer's tests	20 (40)	57	Orbicularis excision during blepharoplasty causes temporary decrease in TBUT and more occurrences of reversible dry eye symptoms
Nalci et al. (2020) [20]	To evaluate the impact of upper eyelid blepharoplasty on CS in dermatochalasis patients	Prospective	Skin-only blepharoplasty	CS measured using sine-wave contrast sensitivity chart, corneal topography	34 (34)	63	CS significantly increased after upper eyelid blepharoplasty, especially at higher spatial frequencies (3, 6, 12, and 18 cpd), which are usually reduced in older adults Keratometric and corneal HOAs did not change significantly
Bhattacharjee et al. (2020) [21]	To analyze the long-term changes in CS and HOAs, and corneal topography after upper eyelid blepharoplasty	Prospective	Excess skin and fat pad excision	CS: Pelli–Robson chart HOAs: WaveLight Allergio analyzer Corneal topography: topographic modeling system-4	30 (60)	56	At 12 months, the mean CS, the majority of HOAs, and corneal topography (only cylinder) showed a stable, statistically significant difference
Ekin and Ugurlu (2020) [22]	To evaluate the changes of VA, CS, astigmatism, and HOA after blepharoplasty	Prospective	Excess skin and fat pad excision	Corneal topography (Sirius)	103 (206)	56.7	No significant differences were observed for VA. The CS significantly increased at all spatial frequencies both under glare and nonglare conditions. The mean refractive astigmatism significantly decreased. In patients with MRD < 2 mm, mean CS was increased and mean astigmatism decreased significantly compared with those with ⩾ 2 mm. HOA and root mean square decreased significantly
Sommer et al. (2022) [4]	To investigate the effect of skin-only upper eyelid blepharoplasty on corneal biomechanics and topographic parameters	Prospective	Excision of redundant skin orbicularis muscle and fat	The corneal resistance factor and corneal hysteresis were assessed by ocular response analyzer Pentacam	35 (42)	64.5	The increasing CH and CRF might indicate a rise of corneal damping capacity. Despite statistically significant differences of Kmax, I-S and ISV, all other tomographical/ topographical parameters did not change. The corneal steepening and the decrease of I-S do not seem to have a clinically relevance

*BCVA* best corrected visual acuity, *HOA* high order aberration, *CS* contrast sensitivity, *MRD* marginal reflex distance

**Table 2:** Summaries of clinical articles reporting the effect of ptosis surgery on corneal biophysics

Author (year)	Aim	Design	Intervention	Methods	Cases (eyes)	Mean age	Outcomes
Brown et al. (1998) [23]	To evaluate corneal curvature changes after ptosis or blepharoplasty surgeries	Prospective	Anterior levator surgery, tarso-myectomy, conjunctiva-mullerectomy, sling	Standard keratometry and Corneal video-keratography	22 (42)	58	Repositioning of the upper eyelid causes visually significant astigmatic change (30% in ptosis group had ≥ 1D change in astigmatism vs 11% in the blepharoplasty group)
Ugurbas and Zilelioglu [8] (1999)	To determine the effect of congenital ptosis on corneal shape and the development of amblyopia	Cross-sectional	-	Topographic Modelling System	22 (44)	15.5	Ptotic eyes had an increased incidence of bow tie pattern astigmatism, corneal asymmetry, and corneal irregularity. Lack of mirror-image symmetry with the fellow eye was higher in amblyopic eyes
Kumar et al. (2013) [24]	To analyze the effect of congenital unilateral ptosis on the ocular HOA	Prospective case series	-	Topography, HOA with Zywave workstation, Schirmer’s test, Break up time and corneal staining	16 (16)	12.5	There was significant difference noted in the mean 6 mm Zernicke coefficients and total RMS between the ptosis and the fellow eyes. Total coma aberration correlated with BCVA and MRD in the ptosis eyes. There was no correlation between the age and total RMS
Ugurbas et al. (2014) [25]	Tear function tests and ocular surface are evaluated in patients who underwent unilateral surgery for mild to moderate ptosis	Prospective	MMCR	Dry eye questionnaire, Schirmer test, TBUT, vital staining, meibomian gland evaluation and conjunctival impression cytology	16 (16)	51	There was no statistically significant change in the tear function tests and goblet cell densities after ptosis surgery
Wee and Lee (2014) [26]	Clinical outcomes of MMCR in patients with mild to moderate ptosis and the effect of CMMR on DED	Retrospective	MMCR	Schirmer test, ocular surface disease index score, phenylephrine test	30 (51)	55.8	CMMR may benefit patients with mild to moderate ptosis and even those with negative phenylephrine test responses. Goblet cell damage can worsen dry eye symptoms
Fowler et al. (2015) [27]	To assess whether taped vs untaped CS testing reliably predicts improvement following eyelid-lifting surgery in patients with ptosis and/or dermatochalasis	Prospective	Levator resection, blepharoplasty	Mars near contrast card held at 40 cm under standard lighting conditions	41 (78)	-	The mean preop untaped CS was 1.30. The mean preop taped and postop log CS were 1.52 (11.85% increase) and 1.51 (11.44% increase), respectively. The difference between the 2 groups was not statistically significant
Agrawal and Ravani (2016) [28]	To document the changes in astigmatism after ptosis correction	Prospective	Fasanellaservat surgery, levator resection and frontalis sling surgery	VA testing, ptosis evaluation, standardized keratometry with Bausch and Lomb keratometer	30	4–12	The average postop change in astigmatism was 0.43 which was statistically significant
Karabulut and Fazil (2019) [29]	To evaluate corneal refractive and topographical changes after MMCR on mild ptosis	Retrospective	MMCR	Corneal topography (Sirius)	28 (28)	31	BCVA and cycloplegic refraction did not change significantly. The mean change in corneal astigmatism, simK, SIf, and CCT did not show significant differences. Apical keratometry front showed a significant decrease at 3 and 6 months
Youssef et al. (2020) [30]	To evaluate corneal topographic changes after ptosis surgery	Prospective	Levator resection/ frontalis sling surgeries	Computerized tomography (Sirius 3D rotating Scheimpflug & topography)	30 (30)	24.7	3 months postop, corneal astigmatism, average keratometry, and apical keratometry front demonstrated a significant reduction The BCVA improved but was statistically insignificant
Gandhi et al. (2020) [31]	To evaluate the effect of frontalis sling surgery for congenital ptosis on corneal curvature and refractive status	Prospective	Frontalis Sling Surgery	Computerized topographer, IOL master, autorefractometer and AS-OCT	48 (60)	18	3 months postop, there was significant reduction of cylindrical by -0.36 D and improvement of BCVA by 0.24 ± 0.04 logMAR. Average keratometry did not change significantly. A greater reduction in astigmatism was noticed in the age group of 5–10 years
Li et al. (2020) [7]	To study the difference in the corneal biomechanical parameters of ptotic and fellow eyes in patients with congenital blepharoptosis	Prospective	Levator resection (LF > 5 mm), frontalis suspension (LF ≤ 5 mm)	LenStar LS900, non-contact tonometer (NCT) and a Corvis ST tonometer	29 (29)	9.7	The Corvis ST parameters (Deformation amplitude [DA], A1 times, and A1 velocity), central corneal thickness (CCT), and IOP with NCT differed significantly between ptotic and fellow eyes. CCT was positively correlated with Length A1 and IOP with Corvis in ptotic eyes
Numata et al. (2021) [32]	To evaluate the corneal topographic changes after ptosis correction with and without deepening of the upper eyelid sulcus	Retrospective	Levator resection surgery	VA: logMAR. Corneal topography was measured using AS-OCT	23 (23)	70	Eyes with deepening of the upper eyelid sulcus blepharoptosis, surgery can change keratometry, cylinder and HOA which can improve the visual function
Mohammed et al. (2021) [28]	To evaluate corneal topographic changes after eyelid ptosis surgery	Prospective	Levator resection	Corneal topography (Sirius)	50	20.7	Flattening of superior cornea shown by significant decrease in apical keratometry front
Assadi et al. (2021) [33]	To evaluate the changes in corneal topography, cycloplegic refraction, and BCVA after ptosis correction surgery in patients with congenital ptosis	Prospective	Isolated congenital ptosis: frontalis sling surgery. Marcus Gunn Jaw Winking Syndrome: LPS disinsertion with frontalis sling surgery	Orbscan 3	21 (27)	11.6	A significant decrease in steepest K was noted postop. Inferior K also decreased significantly. However, change in I-S asymmetry was not significant. Variation in BCVA and cycloplegic sphere and cylinder was minimal. Sim K astigmatism, surface regularity index, I-S asymmetry and central corneal thickness did not show significant variation
Mongkolareepong et al. (2021) [34]	To evaluate the predictive factors of postop corneal astigmatism change after ptosis surgical repair	Retrospective	Congenital: supra maximal levator resection Acquired: levator resection	Nidek Tonoref II Autorefractor Keratometer	28 (42)	16.7	A significant postop corneal astigmatism change was only observed in a subgroup of eyes with preop astigmatism of ≥ 1.5 D. 72.2% of these eyes showed a reduction of astigmatism with a mean change of 0.65 D
Abdel Rahman et al. (2022) [35]	To evaluate the corneal topographic changes after levator resection surgery	Prospective	Transcutaneous levator muscle resection	Topography, Snellen chart and TBUT	20 (20)	6.5	K1, K2, and astigmatism were reduced but not significantly
Ceylan et al. (2022) [36]	To evaluate the effect of upper eyelid surgery on ocular surface and corneal topography	Prospective	Group 1: upper eyelid blepharoplasty Group 2: upper eyelid blepharoplasty and levator advancement ptosis surgery Group 3: levator advancement ptosis surgery	TBUT, ocular surface disease index, Schirmer’s, Autorefractometry and corneal topography (Oculus Pentacam HR)	20 (32)	44	Schirmer test results decreased significantly at 6 months in groups 1 and 2. TBUT values decreased at 1 week in group 3 but returned to baseline at 1 month. Corneal punctate staining was detected at day one and week one in all groups. Group 3 showed a significant change in K2 values at one month

*BCVA* best corrected visual acuity, *HOA* high order aberration, *CS* contrast sensitivity, *RMS* root mean square, *MMCR* Müller’s muscle conjunctival resection

## Main Text

### Corneal optics

The cornea plays a crucial role in visual acuity due to its contribution of approximately two-thirds of the eye’s total optical power. The anterior surface curvature of the cornea is primarily responsible for corneal refraction. Given that this surface is in direct contact with the eyelids, alterations in eyelid shape and function can result in changes to corneal refraction, steep and flat corneal meridians, refractive error, and corneal astigmatism.

Gullstrand’s hypothesis suggests that induced corneal astigmatism is caused by pressure from the eyelid on the vertical meridian, resulting in with-the-rule (WTR) astigmatism [37]. This pressure flattens the corneal periphery while steepening the central region [30, 31, 36]. Consequently, elevating the eyelid causes central flattening and peripheral steepening. Assadi et al. reported significant flattening of the inferior cornea six months after levator resection or sling surgery on 27 eyes with congenital ptosis [33]. Zinkernagel et al. found that all patients showed significant changes in corneal astigmatism within a central 5-mm zone after ptosis correction with a mean change of 0.72 D [14]. Pressure exerted by the eyelid on the periphery of a ptotic eye’s cornea causes a flat periphery and steep center resulting in astigmatism. Ptosis surgery corrects this condition, with the greatest effect being observed at the center of the cornea [14]. Preoperative ptosis severity predicts postoperative corneal astigmatism alteration. Severe ptosis causes steepening of the inferior meridian, which subsequently experiences the greatest flattening after surgery [33]. Conversely, mild ptosis causes steepening of the superior meridian, which then undergoes the most significant flattening postoperatively [29]. Aydemir and Aydemir investigated the impact of ptosis on IOL power calculation. The results indicated that only patients with ptosis greater than 4 mm experienced significant reduction in corneal curvature and astigmatism following surgery [5]. Therefore, for patients with severe eyelid ptosis and cataracts, it is crucial to account for potential changes in IOL power calculation after ptosis surgery.

Uğurbaş and Zilelioğlu found a significantly increased rate of astigmatism in ptotic eyes compared to normal control eyelids in their study on 44 eyes of 22 patients with congenital ptosis. These ptotic eyes typically exhibited a bow-tie pattern on topographic maps [8]. Another cross-sectional study by Kim and Lee on 33,103 Korean individuals reported a hyperopic shift and increased against-the-rule (ATR) astigmatism in ptotic eyelids [3].

A study by Kim and colleagues found that 6 weeks after levator resection surgery, corneal power increased and astigmatism decreased in half of the cases [18]. However, after blepharoplasty surgery, corneal power and astigmatism remained unchanged in most patients. The lower changes in corneal power and astigmatism are attributed to a relatively smaller change in lid position following blepharoplasty surgery. Brown et al. reported average dioptric changes of approximately 0.60 D and 0.55 D after ptosis repair and blepharoplasty surgeries [23]. Nearly 30% of ptosis repair patients exhibited transient astigmatic changes exceeding 1.00 D, compared to only 11% of blepharoplasty patients. The patient may need to change their spectacle or contact lens correction after surgery. Dogan et al. evaluated the corneal parameters using Scheimpflug imaging for patients who underwent upper blepharoplasty [16]. The study found that the steepest keratometry significantly increased in patients with margin reflex distance 1 (MRD1) less than 2.5 mm. However, no significant changes were observed in visual acuity and astigmatic power vectors (J0 and J45). Sommer et al. performed skin-only blepharoplasty on 42 eyes, resulting in a 0.4 D increase in the Kmax after 4 weeks [4]. However, the difference between the inferior-superior (I-S) value decreased by 0.22 D. These changes in corneal curvature did not result in significant changes to the BCVA. Simsek et al. also observed a small mean increase of 0.15 D in corneal astigmatism among 60% of their participants one month after blepharoplasty surgery [9]. However, this increase was only borderline significant three months thereafter. In a study by Zinkernagel and coworkers, 82 eyes were assessed before and 3 months after blepharoplasty or ptosis correction surgery [14]. The authors observed that ptosis correction and blepharoplasty with removal of pre-aponeurotic fat pads caused alterations of 0.25 D and 0.21 D in total corneal astigmatism, respectively. Blepharoplasty without resection of pre-aponeurotic fat pads caused an insignificant change of 0.09 D in corneal astigmatism. This may be due to the lower gravitational effect on the cornea of skin-only blepharoplasty. Interestingly, ptotic patients with deep superior sulcus may experience greater reduction in cylindrical power than others following blepharoptosis surgery due to more structurally rigid eyelids in patients with deepening of the upper eyelid sulcus [32].

Some studies report no significant change in topographic corneal parameters following ptosis correction surgery [28, 35], but these may be limited by small sample sizes or short follow-up periods. Interestingly, Youssef et al. reported a decrease of 0.5 D and 0.9 D in corneal astigmatism and corneal power, respectively, three months after surgery despite finding no significant alteration one month postoperatively [30]. Older studies have reported an increase in corneal astigmatism following ptosis correction surgery, but these primarily used keratometric measurements which do not directly measure corneal curvature. Other studies also reported a reversal of this increase after resolution of temporary post-operative eyelid edema [38]. As post-operative eyelid edema can persist for up to two months following blepharoplasty surgery [39], it is recommended to repeat corneal topography and refraction three months postoperatively before making further decisions.

Mongkolareepong et al. identified pre-operative corneal astigmatism as a key predictor of postoperative change in corneal astigmatism [34]. Specifically, 72.2% of patients with preoperative corneal astigmatism greater than 1.5 D experienced a decrease of 0.65 D following ptosis surgery, while patients with less than 1.5 D corneal astigmatism did not experience significant changes.

Gandhi et al. observed a decrease of 0.47 D in corneal astigmatism following frontalis suspension surgery, with a more pronounced reduction in patients aged 5–10 years [31]. The authors suggested that performing surgery for severe ptosis at a younger age may be more beneficial due to increased corneal flaccidity of children. Another study also documented a significant change of 0.43 diopters in corneal astigmatism three months after ptosis correction surgery in patients aged 4–12 years [40].

Sussenbach et al. described a noteworthy case in which high corneal astigmatism was concealed by the stenopic effect of a ptotic eyelid with a narrow palpebral fissure, resulting in good vision for the patient [41]. However, following surgical elevation of the eyelid, the underlying keratoconus was revealed, and the patient reported a decreased vision.

The factors that influence changes in corneal astigmatism following eyelid surgery can be summarized as follows:

- Type of surgery: Ptosis correction has a greater impact than upper eyelid blepharoplasty, and fat removal during upper blepharoplasty has a greater effect than skin removal alone [10, 11].
- Severity of preoperative ptosis: The more severe the ptosis, the greater the change in corneal astigmatism following surgery [5, 35].
- Preoperative corneal astigmatism: If above 1.5 D, it will have a greater effect in reducing astigmatism after ptosis correction surgery [29].
- Patient age: Younger patients tend to experience a greater reduction in corneal astigmatism following ptosis surgery [18, 23].
- Duration of patient follow-up: Changes in astigmatism become more stable over time after eyelid surgery, particularly after 6 months [9, 14, 30, 33].

Studies have generally shown a mild increase in corneal dioptric power following ptosis repair surgeries. Changes greater than 0.5 D may induce visually significant alterations and should be discussed with the patient prior to surgery [42]. However, other studies have demonstrated that changes in corneal refraction and keratometry six months after levator resection surgery in adults are not statistically significant. Brief subjective visual symptoms may be attributed by changes in the eyelid position or age-related changes in the lens and cataract formation [43].

### Corneal biomechanics

Corneal biomechanics encompasses the properties of the cornea that dictate its response to external forces. The properties are characterized by parameters such as corneal hysteresis and corneal resistance factors. Corneal hysteresis refers to the difference between the air pressure on the cornea during deflation and inflation, while corneal thickness plays a crucial role in determining corneal resistance factors [44]. The impact of ptosis and dermatochalasis on corneal biomechanics is an emerging area of research that has recently garnered attention from several authors. Given that these conditions can affect patients across all age groups, their influence on corneal biomechanics may significantly alter outcomes for various corneal pathologies and treatments, including keratoconus, cataract surgery, and refractive surgery. As such, it is imperative to elucidate the effect of ptosis and dermatochalasis on corneal biomechanics.

In one study conducted by Sommer and colleagues, minimal increases in both corneal hysteresis and corneal resistance factor were observed four weeks following skin-only blepharoplasty surgery; however, these increases were not statistically significant [4]. In another study conducted by Li et al., corneal biomechanical parameters were assessed by Corvis ST tonometry. The results indicated that these parameters differed minimally but significantly between ptotic eyes and normal eyes [7]. Furthermore, these differences remained unchanged six months post-surgery. The authors concluded that ptotic eyes have thicker corneas and exhibit less flexibility in response to external forces. However, further research is necessary to elucidate the potential effects of ptosis and dermatochalasis, as well as their corrective surgeries, on corneal biomechanics.

In brief, it is important to consider that alterations in eyelid position following eyelid surgery may result in significant changes in corneal biomechanical properties. These changes are particularly relevant in corneas with intrinsic pathology or abnormal thickness.

### Contrast sensitivity and higher-order aberrations

Contrast sensitivity is defined as the ability to distinguish an object from its background. This visual ability differs from visual acuity, which measures the clarity of vision at distance. As such, two individuals with identical visual acuity may exhibit different contrast sensitivities that determine their visual performance, particularly in dimly lit environments [45].

Higher-order aberration (HOA) is a significant factor in contrast sensitivity and refers to the distortion acquired by a wavefront of light when it encounters an eye with imperfections in its refractive components. When subjects narrow their palpebral fissures, both HOA and lower-order aberration (LOA) increase [46]. In a study by Han et al., the authors assessed the squeezing effect of the eyelids on the globe in near vision and then administered cycloplegic drops to eliminate their effect [46]. They observed that after treatment with the cycloplegic drug, neither spherical aberration nor spherical defocus increased with squeezing of the eyelids; however, vertical coma and astigmatism were still significantly induced. None of these changes were significant after correction. Accordingly, they concluded that vertical pressure from eyelids when a patient is squeezing them can result in LOA and HOA and cause reduced contrast sensitivity. They also reported that placing artificial eyelashes in front of a subject’s eye increased visual aberrations and reduced vision quality.

In 2015, Fowler et al. published a study assessing the contrast sensitivity of 78 eyes after functional blepharoplasty and ptosis surgery. They reported an 11% increase in contrast sensitivity [27]. Interestingly, this increase was reproducible with preoperative taping of the eyelids in the correct position; the level of contrast sensitivity in the preoperative taped and postoperative periods was not significantly different. In another study, Nalci et al. observed that three months after blepharoplasty for patients with dermatochalasis, contrast sensitivity significantly increased, and this increment was not related to improvement in eyelash ptosis [20]. They reported that this increase mainly occurred in the high spatial frequencies of the contrast sensitivity spectrum; given that this range may decrease with aging, they concluded that blepharoplasty for treatment of dermatochalasis can increase contrast sensitivity and thus improve vision quality in older patients. In their long-term prospective study, Bhattacharjee et al. followed 60 eyes that underwent upper blepharoplasty for a period of 12 months [21]. The authors demonstrated that the mean contrast sensitivity value, HOA, and cylindrical power remained stable and significantly improved. They concluded that older patients undergoing cataract surgery can gain additional visual benefits from upper blepharoplasty. Improvement in photopic and mesopic contrast sensitivity after treatment of dermatochalasis and lash ptosis has been reported in other studies as well [17].

Several reasons have been hypothesized for the increase in contrast sensitivity after blepharoplasty, including treatment of lash ptosis, corneal topographic changes, and reduction of HOA [42]. Redundant skin over the eyelids can reduce light entering the eye and exert a diffractive effect that reduces contrast sensitivity. If dermatochalasis involves the central region of the cornea it can decrease central vision; furthermore, lash ptosis can interfere with central vision [15]. A study conducted by Ekin and Ugurlu in 2019 indicated that contrast sensitivity and HOA significantly improved one month after blepharoplasty [22]. This improvement was more prominent in patients with preoperative MRD1—distance between the upper lid margin and corneal light reflex—lower than 2 mm. Kim et al. also demonstrated that after blepharoplasty, contrast sensitivity and functional vision improved due to reduction of HOA and correcting of eyelash position [2]. The authors reported a decrease in the 3^rd^ and 4^th^ order aberrations as well as coma and trefoil. Kumar et al. reported an increase in total root mean square (RMS) of HOA in the ptotic eyes compared to normal eyes [24]. They also noted significant differences in the mean of multiple Zernicke coefficients at 6 mm.

Briefly, contrast sensitivity significantly increases after blepharoplasty and ptosis correction surgeries (especially in higher spatial frequencies), potentially improving vision quality in patients with severe functional dermatochalasis. This increase in contrast sensitivity can be due to correction of eyelash ptosis and increased light entering the eye, as well as reduction of HOA. This improvement persists for at least three months after surgery; further studies are needed to examine changes beyond this time period [20, 27, 42–46].

### Dry eye

Dry eye symptoms may be exacerbated following blepharoplasty due to several factors including chemosis, postoperative inflammation, lagophthalmos, excess orbicularis resection and/or denervation, eyelid retraction, and malfunction of the lacrimal pumping system [12, 15, 17, 21, 22, 24]. Note that aggressive blepharoplasty leading to lagophthalmos can exacerbate dry eye and lead to keratopathy and neurotrophic keratitis.

A study by Wee and Lee examining 51 eyes investigated the impact of Muller’s muscle-conjunctival resection surgery on dry eye [26]. Evaluation utilizing Schirmer’s test and tear break-up time (TBUT) test revealed that tear adequacy was significantly and transiently disrupted for 2 months following surgery but returned to preoperative levels thereafter. However, Kim et al. found no significant disruption in tear adequacy following either blepharoplasty or ptosis correction surgery [13]. Similarly, other studies did not report this disruption in tear adequacy during long-term follow-up [25, 47]. Interestingly, Rymer et al. demonstrated that dry eye symptoms improved significantly 3 months after blepharoplasty in conjunction with Muller’s muscle conjunctival resection but not in the blepharoplasty-only group [11]. Nevertheless, none of the objective tests such as Schirmer’s test, Rose Bengal staining test, fluorescein staining test or TBUT test showed significant differences between the two groups. The authors postulate that this discrepancy between subjective and objective evaluations may be attributed to an improvement in dermatochalasis and ptosis resulting in patient satisfaction and a reduction in dry eye complaints. Another potential explanation for this observation is that the correction of ptosis may lead to an improvement in blink eyelid excursion, resulting in enhanced distribution of the tear film.

There are several predictive factors that can be used to determine which patients may be susceptible to dry eye symptoms following blepharoplasty surgery. Mohammed observed that a transient reduction in TBUT was seen only when the orbicularis muscle strip was removed during blepharoplasty [19]. Saadat and Dresner also suggested that blepharoplasty without orbicularis strip resection is safe for patients with dry eye disease [12]. Other risk factors for developing or aggravating dry eye after blepharoplasty include preoperative use of lubricating drugs, diabetes, hypothyroidism, a positive history of allergy, and a history of refractive surgery [10, 21]. Dry eye symptoms following blepharoplasty are typically transient and self-limited. McKinney and Byun do not recommend using tear production tests such as Schirmer’s test and TBUT for diagnosing dry eye after blepharoplasty because these tests measure reflex tear production rather than basal excretion [48]. Instead, they suggest that a patient's medical history and careful examination of eyelid anatomy are more accurate predictors of dry eye following eyelid surgery.

In brief, it is important to note that dry eye symptoms may be transiently aggravated during the first few weeks following blepharoplasty or ptosis correction surgery. Patients should be informed of this possibility prior to surgery. Typically, these symptoms will resolve within 2–3 months after eyelid surgery. The risk of induced dry eye can be minimized by performing minimal orbicularis muscle resection and denervation during surgery and by providing intensive treatment of dry eye and blepharitis prior to eyelid surgeries.

### Similarities and differences between blepharoplasty and ptosis surgery

Both procedures can alter ocular properties and functions by modifying retinal lighting and ocular optics. Additionally, both surgeries can exacerbate dry eye symptoms and impact contrast sensitivity, which is a significant factor in visual quality.

However, ptosis correction surgery has a greater impact on corneal astigmatism than upper eyelid blepharoplasty. Fat removal during upper blepharoplasty has a greater effect on corneal astigmatism than skin removal alone. The severity of preoperative ptosis is also a predictor of postoperative corneal astigmatism alteration.

## Conclusions

This review article highlights recent studies that have reported significant effects of upper eyelid surgery on refractive conditions, ocular aberrations, and visual quality. Contrast sensitivity, an important factor role in visual quality, is greatly affected by upper eyelid surgery. Additionally, eyelid surgery can exacerbate dry eye symptoms. However, ptosis correction surgery has been found to have a greater impact on corneal astigmatism than upper eyelid blepharoplasty. Therefore, it is important to consider these factors before undergoing surgery or when planning to have refractive or intraocular procedures after eyelid surgery.

## Data Availability

Not applicable.

## Abbreviations

IOP: Intraocular pressure
IOL: Intraocular lens
ATR: Against-the-rule
MRD1: Margin reflex distance 1
BCVA: Best-corrected visual acuity
WTR: With-the-rule
HOA: Higher-order aberration
LOA: Low-order aberration
RMS: Root mean square
TBUT: Tear break-up time
